# *Pistacia lentiscus* Hydrosol: Untargeted Metabolomic Analysis and Anti-Inflammatory Activity Mediated by NF-*κ*B and the Citrate Pathway

**DOI:** 10.1155/2020/4264815

**Published:** 2020-11-01

**Authors:** Anna Santarsiero, Alberto Onzo, Raffaella Pascale, Maria Assunta Acquavia, Marianna Coviello, Paolo Convertini, Simona Todisco, Michela Marsico, Corrado Pifano, Patrizia Iannece, Carmine Gaeta, Salvatore D'Angelo, Maria Carmela Padula, Giuliana Bianco, Vittoria Infantino, Giuseppe Martelli

**Affiliations:** ^1^Department of Science, University of Basilicata, Viale dell'Ateneo Lucano, 10, 85100 Potenza, Italy; ^2^Bioinnova srls, Via Ponte Nove Luci, 22, 85100 Potenza, Italy; ^3^Dipartimento di Chimica e Biologia, Università degli Studi di Salerno, Via Giovanni Paolo II, 132-84084 Fisciano, Italy; ^4^Rheumatology Department of Lucania, Rheumatology Institute of Lucania (IReL), San Carlo Hospital of Potenza and Madonna delle Grazie Hospital of Matera, Potenza, Italy

## Abstract

*Pistacia lentiscus* shows a long range of biological activities, and it has been used in traditional medicine for treatment of various kinds of diseases. Moreover, related essential oil keeps important health-promoting properties. However, less is known about *P. lentiscus* hydrosol, a main by-product of essential oil production, usually used for steam distillation itself or discarded. In this work, by using ultra-high-resolution ESI(+)-FT-ICR mass spectrometry, a direct identification of four main classes of metabolites of *P. lentiscus* hydrosol (i.e., terpenes, amino acids, peptides, and condensed heterocycles) was obtained. Remarkably, *P. lentiscus* hydrosol exhibited an anti-inflammatory activity by suppressing the secretion of IL-1*β*, IL-6, and TNF-*α* proinflammatory cytokines in lipopolysaccharide- (LPS-) activated primary human monocytes. In LPS-triggered U937 cells, it inhibited NF-*κ*B, a key transcription factor in inflammatory cascade, regulating the expression of both the mitochondrial citrate carrier and the ATP citrate lyase genes. These two main components of the citrate pathway were downregulated by *P. lentiscus* hydrosol. Therefore, the levels of ROS, NO, and PGE_2_, the inflammatory mediators downstream the citrate pathway, were reduced. Results shed light on metabolic profile and anti-inflammatory properties of *P. lentiscus* hydrosol, suggesting its potential as a therapeutic agent.

## 1. Introduction

*Pistacia lentiscus* (*P. lentiscus*) belongs to the *Anacardiaceae* family, consisting of 70 genera and more than 600 species. The genus *Pistacia* includes deciduous resin-bearing shrubs and trees. They are xerophytic trees growing 8–10 m tall. *P. lentiscus*, commonly called mastic tree since its resin is known as mastic, is widely distributed in the Mediterranean basin [1–3]. It is employed in food industry, as a food colorant; in cosmetics, usually sold as essential oil; and for medical purposes, because of its biological activities. Indeed, it is used in the treatment of inflammation, burns, and gastrointestinal complaints [4–8]. Many studies were carried out to uncover the chemical composition of *P. lentiscus* leaves and fruit extracts, by which many constituents were identified and quantified [9–12]. Observed metabolic profile allowed explaining marked antioxidant activity of *P. lentiscus* and several pharmacological activities [11, 13, 14]. Moreover, *P. lentiscus* resin was analyzed by GC-MS, which demonstrated the presence of *α*-pinene, *β*-pinene, limonene, terpene-4-ol, and terpineol [15]. Meanwhile, essential oil obtained from leaves turned out to contain *β*-caryophyllene (31.38%), germaerene (12.05%), and *γ*-cadinene (6.48%). Volatile oil obtained from fruits was analyzed by GC-MS too, thanks to which the presence of *α*-pinene, myrcene and limonene, sesquiterpene, ketones and aliphatic esters, and phenolic compounds (thymol, carvacrol) was appreciated, whereas dimyrcene (0.5-4.4%) was found in all types of oil [16]. Furthermore, many efforts were dedicated to the chemical characterization of *P. lentiscus* essential oil, where the major constituents were terpinen-4-ol, *α*-terpineol, and germacrene D [17–20].

However, by now, less was done for the analysis and characterization of metabolome and biological properties of *P. lentiscus* hydrosol. During distillation for essential oil extraction, a small hydrophilic fraction escapes into the distillation water steam: the result is known as hydrosol. Although several hydrosols are marketed in the Western countries for aromatherapy and other applications, they are mostly considered by-products of steam distillation and, thus, redistilled to obtain more essential oil or discarded thereby losing an economically important coproduct. In the global market, essential oils valued USD 17.2 billion in 2019 and they could reach USD 33.26 billion in the next 8 years [21]. The economic value of the lost oil fraction was estimated to be USD 50-100 million in India [22]. Currently, it would be helpful to understand the potentiality of this low-cost by-product in terms of source of metabolites and therapeutic issues to demonstrate that *P. lentiscus* hydrosol could have potential as a global economic product of commerce.

To elucidate the phytochemical composition of complex matrices, a specific analytical technique is needed such as mass spectrometry, a valuable and mature tool for the assessment of metabolites in different matrices [23–26]. Above all, Fourier transform ion cyclotron resonance mass spectrometry (FT-ICR MS) has been shown to be a powerful technique for obtaining high-resolution accurate-mass spectra with outstanding levels of sensitivity, useful for a correct identification of thousands of unknown substances [27–33]. Thus, in this work, the analysis of *P. lentiscus* hydrosol was performed by using positive electrospray-Fourier transform ion cyclotron resonance mass spectrometry (ESI(+)-FT-ICR MS) together with a specific visualization tool of HRMS data, i.e., the Van Krevelen diagram, to shed some light on its phytochemical composition.

Moreover, since an anti-inflammatory activity of *P. lentiscus* leaf extracts is well-known [34], the influence of *P. lentiscus* hydrosol on proinflammatory cytokines IL-1*β*, IL-6, and TNF-*α* and transcription factor NF-*κ*B was assessed in lipopolysaccharide-triggered macrophages. Then, the focus was set on the citrate pathway, a newly identified target of the immunometabolism under NF-*κ*B control and essential to produce ROS, NO, and PGE_2_ inflammatory mediators [35]. To this end, the effect of *P. lentiscus* hydrosol was evaluated in LPS-triggered U937 cells by quantification of the two main components of the citrate pathway, which are the mitochondrial citrate carrier (CIC) and ATP citrate lyase (ACLY), and the levels of ROS, NO, and PGE_2_ [36, 37].

## 2. Materials and Methods

### 2.1. Plant Material and Extraction

A selected genotype of *P. lentiscus* was applied to perform the different research actions. The genotype used was selected by Bioinnova (Potenza, Italy), starting from an endemism developed in Basilicata region. Young leaves and stems were utilized to obtain the hydrosol. In detail, 500 g of material was submitted to steam distillation for 90 min (with 1.5 L water) using a Clevenger-type apparatus. For each load, fresh material was placed into the distillation vessel. The first load was conducted to set up and establish the procedure and determine the processing parameters, and replications were conducted as the main study. Steam was supplied through a manifold pipe into the bottom of the vessel from a high-pressure boiler. The steam is routed upward through a false-bottom perforated plate to the plant material being extracted. The steam removes the essential oil, and both leave the vessel near the top via a flexible pipe and are carried to a water-cooled, parallel-piped multitubular glass condenser that is mounted vertically. The essential oil (lighter than water) and the water condensate (hydrosol) were then separated in a Pyrex florentine flask that was cleaned prior to and after each use. The yield of the hydrosol fraction obtained considering the initial load of 500 g of material was 0.3 L. Three replications of hydrosol were used to perform all the following experiments. Samples were stored into the fridge at 4°C prior to the analysis.

### 2.2. Mass Spectrometry Analysis

The FT-ICR mass spectrometer used was a Bruker solariX XR Fourier transform ion cyclotron resonance mass spectrometer (FT-ICR-MS) (Bruker Daltonik GmbH, Bremen, Germany) equipped with a 7 T superconducting magnet and an electrospray ionization (ESI) source. The external ESI source was set in positive ionization mode (ESI(+)) and was operated with a grounded capillary sprayer needle mounted 45° off-axis with nitrogen nebulizing gas at a pressure of 1.0 bar, a nitrogen drying gas temperature of 200°C, and a flow rate of 4.0 L/min. The ESI capillary voltage was set to 4.5 KV, while the spray shield voltage was set to -0.5 V. Samples were injected by using a 500 *μ*L syringe (Hamilton, Nevada, USA), setting a syringe flow rate of 120.0 *μ*L/h. Spectra were acquired with a time domain size of 16 megaword, an accumulation time of 0.1 s, and a mass range of 100-2000 *m*/*z*, with an average number of scans of 50. Calibration was performed by using a solution of sodium trifluoroacetic acid (NaTFA) clusters. At least 0.1 ppm mass accuracy FT-ICR MS was achieved. The *P. lentiscus* hydrosol solution filtered by means of a PTFE 0.22 *μ*m filter has been injected directly in the ESI source. An estimation of the noise level was performed by assuming the *N*-sigma methodology [38–40]. A noise level was first estimated by fitting low relative intensity distribution to a Gaussian one, according to the *N*-sigma methodology, in order to calculate its mean. Once obtained, the noise level related to the mass spectrum of *P. lentiscus* hydrosol was set at a relative intensity of 0.5%, i.e., three times the extrapolated mean, in order to eliminate noise peaks, together with artifacts that resulted from Fourier transform execution on obtained free induction decays (FIDs). Moreover, mass spectra were smoothed to further eliminate wiggles and a *m*/*z* list was extracted. Analyses were done in triplicate, and common signals were considered for further data treatment. Then, possible elemental formulae were calculated for each *m*/*z* signal. To obtain unequivocal formulae, several constraints were applied, such as atom number limitations, i.e., C ≤ 100, H ≤ 200, O ≤ 80, N ≤ 20, S ≤ 8, P ≤ 6, Na ≤ 1, and K ≤ 1; restrictions on atoms to carbon number ratios, i.e., 0.2 ≤ H/C ≤ 3.1, O/C ≤ 2, N/C ≤ 1.3, S/C ≤ 0.8, and P/C ≤ 0.3; RDBE > 0; nitrogen rule; and isotopic pattern filtering [41]. Moreover, a Kendrick mass defect analysis was performed to identify homologous series and to help formula assignment by solving redundancies [42–47]. In detail, only *m*/*z* peaks belonging to homologous series with a number of members higher or equal to 2 [45] were taken into account for assignment. The analysis was performed for every selected building block, i.e., CH_2_, CO, H_2_O, H_2_, and O, and results were combined. HRMS data were processed by using Data Analysis (v4.2, Bruker Daltonik GmbH, Bremen, Germany) and R software (v. 3.6.0, https://www.r-project.org/).

### 2.3. Isolation of Human Monocytes from Whole Blood

Primary human monocytes were isolated from blood of healthy donors. The study was made in agreement with the Declaration of Helsinki and in accordance with the Committee on Human Research approved procedure no. 20170030156. All volunteers provided written informed consent approving and authorizing the use of their material for research purposes. Venous blood was collected directly into K2 EDTA-coated BD vacutainer tubes (Becton, Dickinson and Company, Franklin Lakes, NJ, USA). Peripheral blood mononuclear cells (PBMCs) were separated by Histopaque-1077 (Sigma-Aldrich, St. Louis, MO) density gradient centrifugation. In brief, whole blood was mixed with Hanks' Balanced Salt Solution (HBSS, Sigma-Aldrich) at a ratio of 1 : 2 (*v*/*v*), layered on the top of Histopaque-1077 (Sigma-Aldrich) and centrifuged at 1000 × g for 15 minutes. The mononuclear cells at the interphase were recovered, washed twice in HBSS, and counted. PBMCs were incubated with CD14 antibody conjugated to magnetic beads (MACS®, Miltenyi Biotec GmbH, Bergisch Gladbach, Germany) for 15 minutes at 4°C. After washing, cells were loaded onto a MACS® column (Miltenyi Biotec) placed in a magnetic field and CD14-positive (CD14^+^) and CD14-negative (CD14^−^) populations were separated. The CD14^+^ monocytes were cultured in Roswell Park Memorial Institute (RPMI) 1640 medium supplemented with 10% fetal bovine serum, 2 mM L-glutamine, 100 U/mL penicillin, and 100 *μ*g/mL streptomycin at 37°C in 5% CO_2_.

### 2.4. Cell Culture and Treatments

Human monoblastic leukemia U937 cells (ICLC HTL94002-Interlab Cell Line Collection) were grown in suspension in RPMI 1640 medium supplemented with 10% fetal bovine serum, 2 mM L-glutamine, 100 U/mL penicillin, and 100 *μ*g/mL streptomycin in a humidified chamber with 5% CO_2_ at 37°C. Promonocytic U937 cells were differentiated to macrophages by using 10 ng/mL phorbol 12-myristate 13-acetate (PMA, Sigma-Aldrich) and further stimulated with lipopolysaccharide from *Salmonella enterica* serotype typhimurium (LPS, Sigma-Aldrich).

### 2.5. Cytotoxicity Assays

The effect of *P. lentiscus* hydrosol (*P.l*) on cell viability was determined by MTT assay and by cell counting. CD14^+^ monocytes and U937 cells were seeded into a 96-well plate (1 × 10^4^ cells/well) and treated with *P.l* at two different dilutions, 1 : 10 (*P.l* 1 : 10) and 1 : 100 (*P.l* 1 : 100), for 72 hours. MTT assay (*CellTiter 96® Non-Radioactive Cell Proliferation Assay*, Promega, Madison, WI, USA) was performed according to the manufacturer's instructions. In brief, a premixed dye solution was added. During 4-hour incubation, living cells converted a MTT tetrazolium component into formazan. Then, by adding Solubilization/Stop Solution, formazan crystals were solubilized and the absorbance at 570 nm was revealed by a microplate reader (GloMax, Promega). Cell counting was carried out by using the automated handheld *Scepter 2.0 Cell Counter* (Merck Millipore, Switzerland).

### 2.6. Quantification of Cytokines

The CD14^+^ monocytes were treated with *P.l* 1 : 10 and *P.l* 1 : 100 for one hour, and then LPS was added to trigger the inflammatory cascade. Twenty-four hours later, the cell culture supernatants were collected and assayed for the concentration of IL-1*β* (DLB50), IL-6 (D6050), and TNF-*α* (DTA00D) by using ELISA kits (R&D Systems, Inc., Minneapolis, MN, USA) following the manufacturer's recommendations.

### 2.7. SDS-PAGE and Western Blotting

For immunoblot analysis, cells were lysed in Laemmli buffer and boiled at 100°C for 5 minutes. Thirty micrograms of proteins was subjected to SDS/PAGE electrophoresis on 7–12% SDS polyacrylamide gels and then electroblotted onto nitrocellulose membranes. The membranes were blocked for 1 hour in a Tris-buffered saline (TBS) solution containing 5% nonfat dry milk and 0.5% Tween 20 and then incubated at 4°C overnight with anti-NF-*κ*B/p65 (ab7970, Abcam, Cambridge, MA), anti-CIC [35, 36], anti-ATP citrate lyase (ab157098, Abcam), or anti-*β*-actin (ab8227, Abcam) antibodies. Following 1-hour incubation with HRP goat anti-rabbit IgG antibody (Santa Cruz Biotechnology, Santa Cruz, CA, USA), the immunoreactions were detected by using the horseradish peroxidase substrate *WesternBright™ ECL* (Advansta, Menlo Park, CA, USA).

### 2.8. Transient Transfection

To measure *ACLY* gene promoter activity, U937/PMA cells were transiently transfected as described previously [48] using 0.5 *μ*g of pGL3 basic-LUC vector (Promega) containing the −3116/−20 bp region of the *ACLY* gene promoter or a deletion fragment of this region. Cells were transfected also with 10 ng of pRL-CMV (Promega) to normalize the extent of transfection. Twenty-four hours after transfection, U937/PMA cells were triggered with LPS in the presence or absence of *P.l* 1 : 10 or *P.l* 1 : 100. The day after, cells were lysed and assayed for LUC activity using the *Dual-Luciferase® Reporter Assay System* (Promega), according to the manufacturer's protocol. Luminescence was measured as relative light units (RLU), taking the reading of luciferase assay substrate alone and then with cell lysate in a GloMax plate reader (Promega).

### 2.9. ACLY Activity

U937/PMA cells were treated for 3 hours with LPS in the presence or absence of *P.l* 1 : 10 or *P.l* 1 : 100; then, cells were washed twice in ice-cold PBS. The cell pellet was resuspended in ice-cold 0.1% NP40 in PBS, and three freeze-melt cycles (-80°C for 8 minutes/40°C for 4 minutes) were performed. After centrifugation, the supernatant was collected and protein concentration was determined by Bradford assay [49]. ACLY activity was assessed by the coupled malic dehydrogenase method [50–52]. In a cuvette, 150 *μ*g of the cell lysate was added to the mixture, made of 50 mM Tris-HCl (pH 8.0), 10 mM MgCl_2_, 1.9 mM DTT, 0.15 mM NADH, 0.07 mM CoA, 1 mM ATP, 2 mM potassium citrate, and 3.3 units/mL malic dehydrogenase. The reaction was started by adding ATP, and the NADH oxidation was measured at 340 nm at 25°C with a spectrophotometer. The specific ACLY activity was determined by normalization to the protein concentration and expressed as a percentage of the control.

### 2.10. ROS, NO, and PGE_2_ Detection

To evaluate reactive oxygen species (ROS) and nitric oxide (NO) levels, U937 cells were differentiated to macrophages with PMA (U937/PMA) and triggered by LPS in the presence or not of *P.l* 1 : 10 or *P.l* 1 : 100. Following 24 hours, ROS and NO concentrations were measured by using 6-carboxy-2′,7′-dichlorodihydrofluorescein diacetate (DCF-DA, Thermo Fisher Scientific, San Jose, CA, USA) and 4-amino-5-methylamino-2′,7′-difluorofluorescein diacetate (DAF-FM Diacetate, Thermo Fisher Scientific), respectively. Cells were incubated with DCF-DA or DAF-FM diacetate for 30 minutes in the dark, and the fluorescence was recorded using a 96-well plate reader (GloMax, Promega). The levels of prostaglandin E_2_ (PGE_2_) were quantified in the cell culture supernatant after 48 hours of exposure to LPS by using *DetectX® Prostaglandin E2 High Sensitivity Immunoassay Kit* (Arbor Assays, Ann Arbor, MI, USA) according to the manufacturer's protocol.

### 2.11. Statistical Analysis

Results are shown as means ± S.D. of, at least three independent experiments. Statistical significance of differences was determined by using one-way ANOVA followed by Tukey's *post hoc* test for multiple comparisons. Differences were considered significant (*p* < 0.05), very significant (*p* < 0.01), and highly significant (*p* < 0.001).

## 3. Results

### 3.1. ESI(+)-FT-ICR MS Analysis

**Figure 1:**
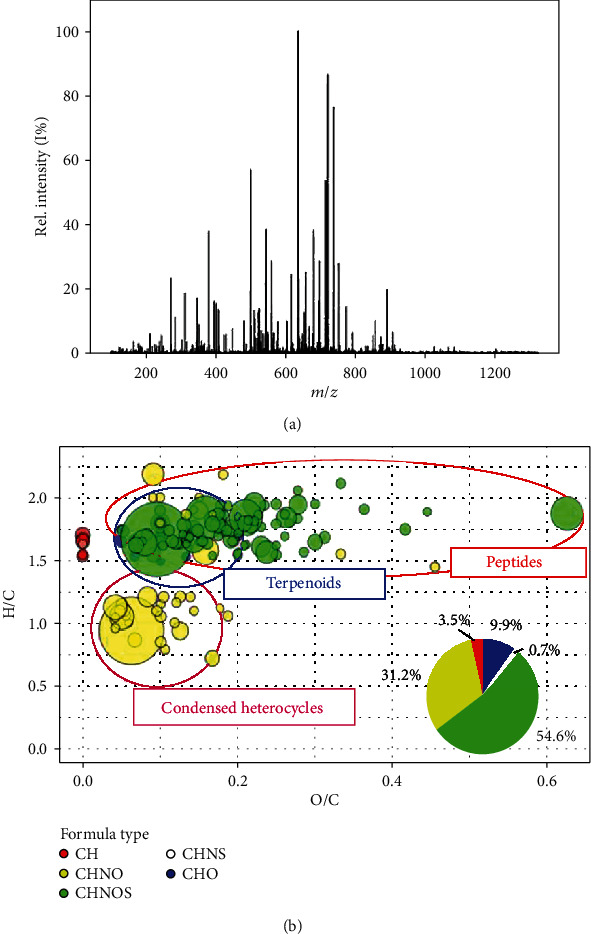
Untargeted metabolomic analysis of *P. lentiscus* hydrosol: (a) ultra-high-resolution ESI(+)-FT-ICR mass spectrum of a sample of *P. lentiscus* hydrosol; (b) Van Krevelen diagram of a sample of *P. lentiscus* hydrosol. Formula types are distinguished by colors, i.e., blue for *CHO*, yellow for *CHON*, green for *CHONS*, pink for *CHNS*, and red for *CH*. Moreover, a pie chart shows percentages of formulae per type.

The ultra-high-resolution ESI(+)-FT-ICR mass spectrum of a sample of *P. lentiscus* hydrosol was obtained (Figure 1(a)), which immediately reveals its high complexity in terms of metabolomic profile. Unfortunately, due to this, it is really hard to unbox to obtain important information related to its chemical composition. For this reason, utilization of specific visualization tools is compulsory. However, several pretreatment steps must be followed in order to eliminate noise peaks and artifacts [38–40]. 141 unequivocal formulae were assigned to leftover signals by considering several constraints and by assuming the Kendrick mass defect approach (see Materials and Methods), and these were used to build a Van Krevelen diagram, a well-known visualization tool over which molecular formulae are spread depending on their H/C (*Y*-axis) and O/C (*X*-axis) ratios (Figure 1(b)) [41]. This plot is extremely useful to shed some light on the metabolic profile of *P. lentiscus* hydrosol. Indeed, metabolites can be classified according to their position on the plot. In this way, the presence of four main classes of compounds could be appreciated, i.e., terpenes, amino acids, peptides, and condensed heterocycles. To be more specific, *CHO*-type formulae located in the upper part of the Van Krevelen diagram suggest the presence of terpenoid compounds in our sample. In detail, obtained formulae could correspond to molecules like verbenone and pinocarvone (C_10_H_14_O), linalool and borneol (C_10_H_18_O), *β*-phellandrenol and myrthenol (C_10_H_16_O), and sobrerol and p-menth-2-ene-1,8-diol (C_10_H_18_O_2_), already found in hydro- and hydroalcoholic extracts of *P. lentiscus* in high quantities [5, 53]. Moreover, additional higher RDBE *CHO*-type formulae are present into the molecular map, which support the presence of higher RDBE lipid derivatives, like phenolic lipids. All these kinds of compounds are well-known for their affirmed biological activity [54].

By the way, it is interesting to note that there is a higher density of points in other regions of the *Van Krevelen* plot, thus revealing the presence of other types of metabolites never reported for *P. lentiscus*. In detail, the percentage of *CHON* and *CHONS* formulae (Figure 1(b)) is higher, thus suggesting a higher frequency of nitrogen- and sulphur-bearing compounds. From the position of points related to nitrogen-bearing class of formulae into the molecular map, it can be noticed that these species belong to two specific classes, i.e., peptides and condensed heterocycles, like purine and indole derivatives. Among these, amino acids and peptides deserve a special attention; in fact, most of them showed marked health-promoting properties [55–60]. Thus, analysis of high-resolution mass spectrometry data, together with the utilization of specific visualization tools, shed light on the wider complexity of *P. lentiscus* hydrosol metabolic profile, a characteristic that could turn out in a marked biological activity of our sample [61, 62].

### 3.2. *P. lentiscus* Hydrosol Affects the Secretion of the Proinflammatory Cytokines IL-1*β*, IL-6, and TNF-*α*

Since the leaf extracts of *P. lentiscus* have shown strong ability to reduce the levels of IL-6 and TNF-*α* in LPS-triggered polymorphonuclear cells [34], we decided to evaluate the anti-inflammatory activity of *P. lentiscus* hydrosol beginning from the analysis of its effect on proinflammatory cytokines. To this end, human CD14^+^ monocytes were treated with lipopolysaccharide, a structural component of the outer membrane of Gram-negative bacteria able to induce a strong inflammatory response, mediated by the toll-like receptor 4 (TLR4) [63]. LPS induces a wide range of responses in monocytes, including the rapid synthesis of the proinflammatory cytokines TNF-*α*, IL-1*β*, and IL-6 [64].

**Figure 2:**
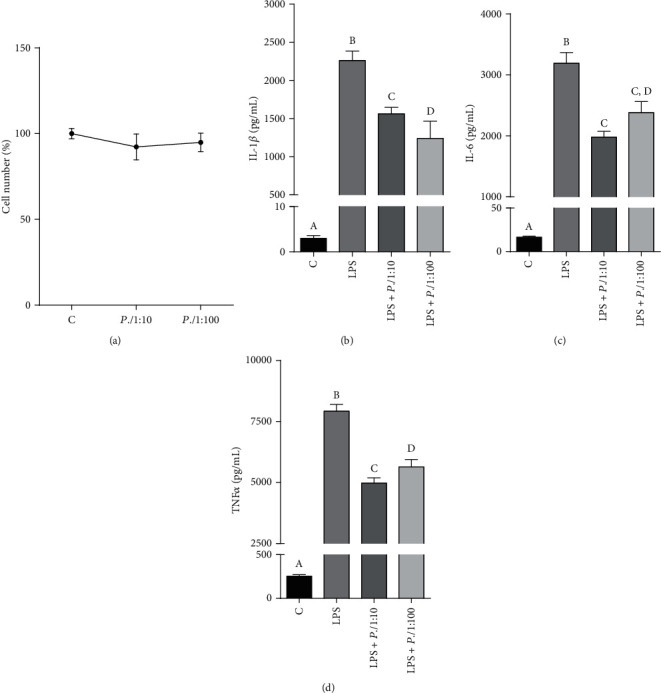
*P. lentiscus* hydrosol affects the release of the proinflammatory cytokines IL-1*β*, IL-6, and TNF-*α*. (a) Primary human CD14^+^ monocytes were treated with *P.l* 1 : 10 and *P.l* 1 : 100 to evaluate the cytotoxic effect of *P. lentiscus* hydrosol. Cell viability was assessed by cell count after 72-hour exposure. Means ± S.D. of three replicate independent experiments are shown. Differences between treated and untreated cells (C, set at 100%) were not significant (one-way ANOVA). (b–d) CD14^+^ monocytes were incubated for 1 hour with *P.l* 1 : 10 and *P.l* 1 : 100 and then activated with LPS. Twenty-four hours later, the concentration of the proinflammatory cytokines IL-1*β* (b), IL-6 (c), and TNF-*α* (d) in cell culture supernatants was measured by ELISA. The values are presented as the mean ± S.D. of three independent experiments. According to one-way ANOVA, differences in IL-1*β* (b), IL-6 (c), and TNF-*α* (d) levels were significant (*p* < 0.001). Therefore, Tukey's post hoc test was performed and different letters indicate significant differences between treatments at *p* < 0.05.

Firstly, we evaluated the cytotoxicity of *P. lentiscus* hydrosol. To this end, human CD14^+^ monocytes, isolated from peripheral blood, were treated with this hydrosol at two different dilutions, 1 : 10 (*P.l* 1 : 10) and 1 : 100 (*P.l* 1 : 100). After 72 hours, cell counting and MTT assay were performed. As shown in Figure 2(a), at tested concentrations, the hydrosol had no obvious effect on the number of viable cells (one-way ANOVA). Similar results were obtained by using MTT assay (Figure S1). Therefore, for all subsequent experiments aimed at evaluating the anti-inflammatory activity, *P. lentiscus* hydrosol was used at 1 : 10 and 1 : 100 dilutions.

In primary human monocytes, IL-1*β*, IL-6, and TNF-*α* secretion after stimulation with LPS was assessed with and without coincubation with *P.l* 1 : 10 or *P.l* 1 : 100. More in detail, human CD14^+^ monocytes were treated for 1 hour with *P. lentiscus* hydrosol; then, LPS was added; 24 hours later, the concentration of IL-1*β*, IL-6, and TNF-*α* was quantified in the cell culture supernatant. We observed a marked increase in the levels of all the proinflammatory cytokines analyzed after induction with LPS (Figures 2(b)–2(d): LPS *vs.* C, *p* < 0.001, Tukey's test). *P. lentiscus* hydrosol lowered IL-6 and TNF-*α* secretion in a dose-dependent manner, while *P.l* 1 : 100 brought the concentration of IL-1*β* down more than *P.l* 1 : 10 (Figures 2(b)–2(d)). In particular, *P.l* 1 : 100 reduced almost half the levels of IL-1*β* released after stimulation with LPS (Figure 2(b): LPS+*P.l* 1 : 100 *vs.* LPS, *p* < 0.001, Tukey's test), whereas the decrease caused by *P.l* 1 : 10 was about 35% (Figure 2(b): LPS+*P.l* 1 : 10 *vs.* LPS, *p* < 0.001, Tukey's test). On the other hand, *P.l* 1 : 10 led a 4-fold reduction in either IL-6 and TNF-*α* levels (Figures 2(c) and 2(d): LPS+*P.l* 1 : 10 *vs.* LPS, *p* < 0.001, Tukey's test); meanwhile, *P.l* 1 : 100 lowered the latter two cytokines by only 25% with respect to cells triggered only with LPS (Figures 2(c) and 2(d): LPS+*P.l* 1 : 100 *vs.* LPS, *p* < 0.01, Tukey's test).

### 3.3. *P. lentiscus* Hydrosol Reduces NF-*κ*B and Mitochondrial Citrate Carrier Expression

The following experiments were performed on differentiated U937 cells (U937/PMA), which exert a myriad of macrophage functions and mimic the inflammatory response of activated macrophages when stimulated with LPS [65]. First of all, we checked that *P. lentiscus* hydrosol was not toxic also for U937 cells by cell counting (Figure S2a) and MTT assay (Figure S2b). Cell viability was not significantly altered after 72 hours of treatment with *P.l* 1 : 10 or *P.l* 1 : 100 (Figure S2). Therefore, *P. lentiscus* hydrosol was used at those two dilutions for all subsequent experiments on U937 cells.

**Figure 3:**
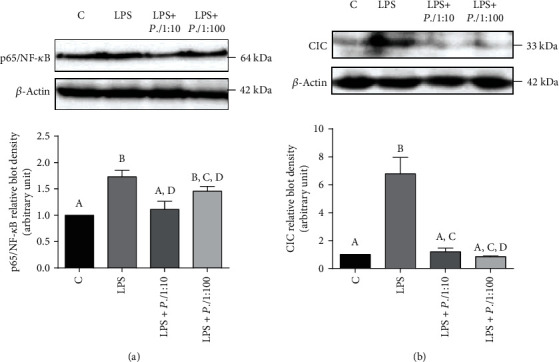
*P. lentiscus* hydrosol reduces p65/NF-*κ*B and CIC expression. U937 cells were treated with *P.l* 1 : 10 or *P.l* 1 : 100 for 1 hour and then activated with LPS. Cell lysates, obtained also from untreated cells (C) and cells triggered only with LPS (LPS), were used to evaluate the expression levels of p65/NF-*κ*B (a) and CIC (b). p65/NF-*κ*B, CIC, and *β*-actin were detected by specific antibodies, and Western blotting images (upper panel (a, b)) are representative of two independent experiments with similar results. The intensities of immunolabeled protein bands were measured by a quantitative software and normalized to *β*-actin (lower panel (a, b)). Bar charts reported means ± S.D. of p65/NF-*κ*B (a) and CIC (b) protein levels obtained from the abovementioned two independent experiments. Different letters indicate significant differences between treatments at *p* < 0.05 (Tukey's test).

The primary focus was on NF-*κ*B, which has a key role in the transcriptional activation of proinflammatory genes in LPS-triggered inflammation [66]. Interestingly, *P. lentiscus* hydrosol was able to resolve LPS-induced inflammation by reducing the levels of NF-*κ*B. In detail, U937 cells differentiated to macrophages with PMA were preincubated for 1 hour with *P.l* 1 : 10 or *P.l* 1 : 100. After that, U937/PMA cells were triggered with LPS. Following 6-hour incubation at 37°C, LPS induced a marked activation of NF-*κ*B p65 subunit (Figure 3(a)). Of note, *P. lentiscus* hydrosol reduced NF-*κ*B p65 subunit expression levels with respect to LPS-triggered cells (LPS) when used as 1 : 100 and at even lower values if employed at the highest concentration (1 : 10) (Figure 3(a)).

As shown above [67], among the proinflammatory genes activated by NF-*κ*B, there is *SLC25A1*, encoding the mitochondrial citrate carrier (CIC). CIC is a member of the mitochondrial carrier family (SLC25) localized in the inner membrane of mitochondria. SLC25s encode membrane proteins that transport many solutes across the inner mitochondrial membrane linking mitochondrial and cytosolic processes thus representing a wide integrated system interplaying with pathologies, such as inflammation or cancer [48, 68, 69]. For this reason, SLC25 gene expression is tightly regulated in physiological and pathological conditions as well as in different tissues and development [70–77]. Moving citrate from mitochondria to the cytosol, CIC is a newly identified target of the immunometabolism and its function is essential to produce inflammatory mediators. Indeed, a great *SLC25A1* upregulation occurs in M1 macrophages [36, 67].

Our analysis confirmed this change in gene expression since 7-fold increase in CIC protein levels was observed after 6 hours of exposure to LPS in U937/PMA cells (Figure 3(b)). *P. lentiscus* hydrosol totally abolished the CIC increased activation induced by LPS whether used as 1 : 10 or 1 : 100 (Figure 3(b)).

### 3.4. Effect of *P. lentiscus* Hydrosol on ATP Citrate Lyase

**Figure 4:**
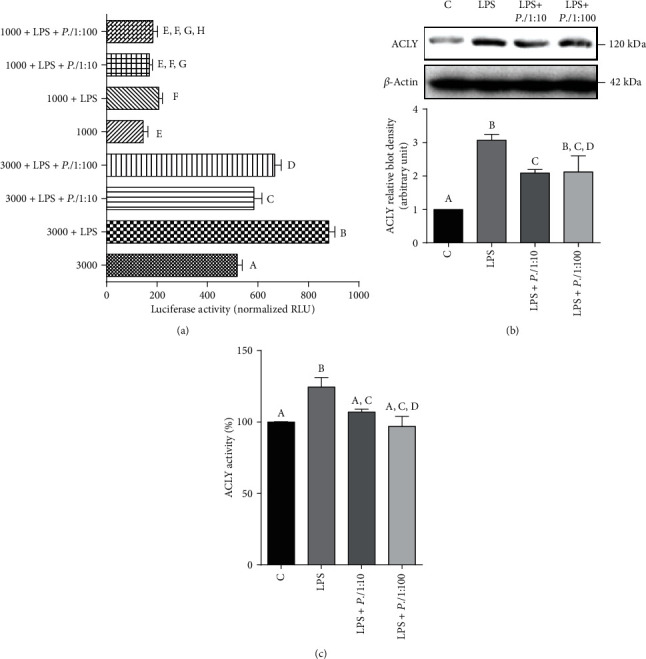
Effect of *P. lentiscus* hydrosol on ATP citrate lyase. (a) U937 cells were transiently transfected with pGL3 basic-LUC vector containing the full-length −3116/−20 bp region of the *ACLY* gene promoter (3000) or a truncated version of this region (1000). Then, cells were triggered with LPS in the absence (LPS) or in the presence of *P.l* 1 : 10 or *P.l* 1 : 100. Unstimulated cells were used as negative control. Twenty-four hours later, the luciferase gene reporter activity was assessed. (b, c) U937 cells, preincubated for 1 hour with *P.l* 1 : 10 or *P.l* 1 : 100, were activated with LPS, and ACLY protein levels (b) and enzymatic activity (c) were evaluated. In (b), ACLY and *β*-actin proteins were immunodecorated with specific antibodies. The Western blotting image (upper panel (b)) is representative of two independent experiments with similar results. The intensities of immunolabeled protein bands were measured by using a quantitative software and normalized to *β*-actin (lower panel (b)). The bar chart reported means ± S.D. of ACLY (b) protein levels obtained from the abovementioned two independent experiments. In (a) and (c), activities are shown as the mean ± S.D. of three experiments. Statistical significance of differences was evaluated by one-way ANOVA followed by Tukey's test for multiple comparisons. Different letters indicate significant differences between treatments at *p* < 0.05.

Since *P. lentiscus* hydrosol inhibited *SLC25A1* gene expression, we wondered if *ACLY*—the gene encoding the enzyme immediately downstream to CIC—could be a target too. ACLY is early upregulated in macrophages activated by LPS or proinflammatory cytokines TNF-*α* or IFN*γ* as well as in inflammatory conditions [37, 78, 79]. It is known that NF-*κ*B transcription factor regulates the expression of ACLY in LPS-triggered macrophages [37]. Because the human *ACLY* gene promoter contains an active NF-*κ*B responsive element (−2048/−2038 bp), U937/PMA cells were transiently transfected with pGL3 basic-LUC vector containing the full-length −3116/−20 bp region of the *ACLY* gene promoter (3000, Figure 4(a)) or a truncated version of this region (1000, Figure 4(a)). First of all, lower *ACLY* gene promoter activity was observed in cells transfected with 1000 than 3000, which contains the binding site for NF-*κ*B, confirming that *ACLY* is under the transcriptional control of NF-*κ*B (Figure 4(a)). Following 24 hours of LPS treatment, the strongest promoter activity was registered in 3000 transfected U937 cells (3000+LPS, Figure 4(a)). The luciferase gene reporter activity was significantly reduced by 30% in cells treated with *P.l* 1 : 10 or *P.l* 1 : 100 (3000+LPS+*P.l* 1 : 10/1 : 100 *vs.* 3000+LPS, *p* < 0.001, Tukey's test, Figure 4(a)). *P. lentiscus* hydrosol was able to induce a parallel decrease in ACLY protein levels and enzymatic activity in LPS-triggered U937 (Figures 4(b) and 4(c)). More in detail, LPS induced a threefold increase in ACLY expression levels (Figure 4(b)) and a 30% rise in ACLY activity (Figure 4(c)). *P. lentiscus* hydrosol led to a reduction in ACLY protein levels in a dose-dependent manner (Figure 4(b)) while no significant differences were observed between *P.l* 1 : 10 and *P.l* 1 : 100 in bringing ACLY activity down (Figure 4(c)). All these results demonstrate that the inhibition of ACLY by *P. lentiscus* hydrosol in LPS-activated U937 cells is mediated by NF-*κ*B.

### 3.5. *P. lentiscus* Hydrosol Reduces ROS, NO, and PGE_2_ Levels Acting through the Citrate Pathway

**Figure 5:**
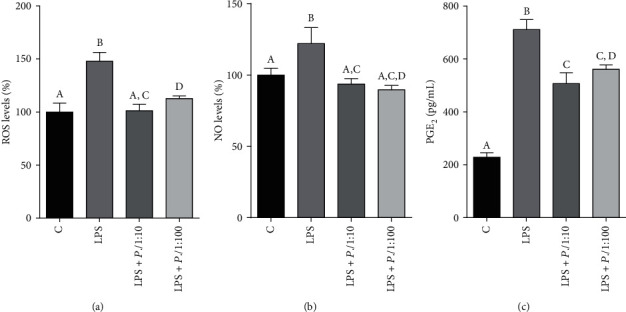
*P. lentiscus* hydrosol lowers ROS, NO, and PGE_2_ levels. U937/PMA cells were untreated (C) or activated with LPS alone (LPS) or in the presence of *P.l* 1 : 10 or *P.l* 1 : 100. (a, b) Following 24 hours, ROS and NO levels were evaluated and expressed as percentage of C (set at 100%). (c) The concentration of PGE_2_ secreted into cell culture supernatants was measured after 48 hours of treatment. Means ± S.D. of four replicate independent experiments are shown. According to one-way ANOVA, differences in ROS (a), NO (b), and PGE_2_ (c) levels were significant (*p* < 0.001). Therefore, Tukey's post hoc test was performed and different letters indicate significant differences between treatments at *p* < 0.05.

To shed more light on the role of *P. lentiscus* hydrosol in regulating the citrate pathway in LPS-activated macrophages, we took into consideration that CIC and ACLY supply intermediaries for the biosynthesis of inflammatory mediators such as ROS, NO, and prostaglandin E_2_ [35]. Therefore, an enhanced release of ROS, NO, and PGE_2_ was observed when U937/PMA cells were treated with LPS (Figures 5(a)–5(c)). *P. lentiscus* hydrosol was able to restore normal levels of reactive oxygen species and nitric oxide at both tested concentrations (Figures 5(a) and 5(b)). Instead, a dose-dependent reduction in PGE_2_ levels occurred: *P.l* 1 : 10 induced a threefold decrease while *P.l* 1 : 100 lowered PGE_2_ concentration only twice with respect to U937/PMA cells triggered with LPS (LPS+*P.l* 1 : 10/1 : 100 *vs.* LPS, *p* < 0.001, Tukey's test, Figure 5(c)).

## 4. Discussion

This study improves the knowledge on metabolites occurring in *P. lentiscus* hydrosol, thus revealing the wide diversity of its metabolic profile. The results of the present study also highlight the health-promoting value of *P. lentiscus* hydrosol. Of note, the sample showed a marked anti-inflammatory activity, most probably due to the presence of specific classes of metabolites, appreciated by the untargeted high-resolution mass spectrometric analysis. More specifically, the Van Krevelen diagram supported the presence of three main classes of metabolites, whose members are already known to show a marked anti-inflammatory activity. In detail, the presence of a considerable amount of anti-inflammatory peptides in different plant species was already demonstrated [55–57]. Moreover, most of the observed peptides are sulphur-bearing ones, thus suggesting the presence of cysteine and methionine units in their structures. It is well-known that the presence of cysteine units in some peptide structures, such as the glutathione (GSH) system, is compulsory to conduct an antioxidant activity against reactive oxygen species and nitric oxide [58–60]. Indeed, these species act synergically through oxidation and formation of disulphide bonds to reduce these harmful reactive species.

The sample tested was safe because of its null toxicity likewise all hydrosols. *P. lentiscus* hydrosol reduced the secretion of the proinflammatory cytokines IL-1*β*, IL-6, and TNF-*α*, which have a key role in the immune response regulation. Notably, preliminary results of an ongoing investigation focused on Behçet's syndrome (BS), a multisystemic inflammatory disorder [80, 81], shows a similar effect on cytokine IL-1*β*.

Moreover, the ability of *P. lentiscus* hydrosol to modulate the activation of NF-*κ*B should be emphasized since NF-*κ*B is a nuclear transcription factor, which regulates the expression of most genes crucial in driving the inflammatory response. Among the NF-*κ*B target genes, we have chosen to focus on *SLC25A1* and *ACLY* immunometabolic genes encoding the mitochondrial citrate carrier and ATP citrate lyase, respectively. Indeed, *SLC25A1* and *ACLY* expression—transcriptionally regulated by NF-*κ*B—strongly decreased following the treatment with *P. lentiscus* hydrosol of LPS-triggered macrophages. Both CIC and ACLY proteins play a crucial role in M1 macrophages by exporting the accumulated citrate from mitochondria to the cytosol and converting it into oxaloacetate (OAA) and acetyl-CoA. OAA produces NADPH through the downstream reactions catalyzed by the cytosolic malate dehydrogenase (MDH1) and the malic enzyme 1 (ME1) [82]. This citrate-derived NADPH represents a critical source of reducing equivalents required for ROS and NO synthesis, as *ACLY/SLC25A1* gene silencing as well as their activity inhibition strongly reduces ROS and NO production in LPS-activated macrophages [35–37, 67, 83]. Moreover, NADPH or malate addition reverts ACLY inhibition phenotype leading to a huge increase of both inflammatory mediators in lymphoblasts [78]. Therefore, the effect of *P. lentiscus* hydrosol on ROS and NO levels could occur through the citrate pathway suppression together with a direct inhibition of NF-*κ*B which controls the expression of *SLC25A1* and *ACLY* but also of NADPH oxidase and inducible NO synthase genes involved in ROS and NO synthesis, respectively [84, 85].

Acetyl-CoA—the second citrate-derived metabolite—is a substrate for lipid biosynthesis including arachidonic acid, essential for the production of prostaglandins. Among them, prostaglandin E_2_ is a key modulator of inflammation and innate immunity and plays a crucial role in inflammatory diseases [86]. Notably, treatment with exogenous acetate nearly entirely reverts the reduction of PGE_2_ levels induced by citrate export pathway inhibition in M1 macrophages [67]. Thus, the lowering PGE_2_ secretion observed in the presence of *P. lentiscus* hydrosol may be the effect of CIC and ACLY decreased expression and activity. Therefore, *P. lentiscus* hydrosol, in addition to diminishing the known IL-1*β*, IL-6, and TNF-*α* proinflammatory cytokines, affects immunometabolism by suppressing the citrate export pathway through NF-*κ*B.

NF-*κ*B inhibition exploited by *P. lentiscus* hydrosol could be explained in terms of the metabolic profile of the sample. Indeed, ultra-high-resolution mass spectrometry data supported the presence of compounds that are known already to inhibit the activation of NF-*κ*B, such as linalool and *α*-terpineol [87, 88]. Moreover, sulphur-containing compounds could play a role into the inhibition process too, as already seen for cysteine-containing peptides and derivatives. For example, N-acetyl cysteine was seen to suppress the activation of inhibitory protein I*κ*B kinases, thus inhibiting NF-*κ*B activation indirectly, and cysteine and glutathione levels turned out to be strictly correlated to the TNF-induced NF-*κ*B activity in cultured mouse hepatocytes [58, 89].

The achieved results are consistent and encouraging since they show for the first time the anti-inflammatory potential and envisage a therapeutic use of *P. lentiscus* hydrosol that until now has been discarded as it was considered a by-product. The exploitation of *P. lentiscus* hydrosol could represent a sustainable approach, according to the circular economy principles, to face the environmental issues derived by the generation of massive amount of these wastes. In fact, hydrosols are easy and inexpensive to produce since they are obtained at the same time of essential oil. Furthermore, in compliance with the circular economy concept, *P. lentiscus* hydrosol must be more investigated and valorized. Hydrosols find great application in aromatherapy for several reasons (*e.g.*, antibacterial, antifungal, antiseptic, analgesic, antioxidant, anti-inflammatory, digestive, healing, and calming properties) [90–93]. Therefore, the study and the discovery of other *P. lentiscus* hydrosol activities could be helpful to drive toward an intensive use of this by-product. Moreover, further studies are needed to isolate, characterize, and elucidate the structure of the bioactive compounds of this sample, to understand which one of them is more correlated to its biological activity, and to be able to develop promising nutraceutical formulations.

## 5. Conclusions

For the first time, this study pointed out on the complex metabolic profile of *P. lentiscus* hydrosol, considered until now a by-product and, so, discarded. Its anti-inflammatory proprieties were highlighted. *P. lentiscus* hydrosol was able to reduce the secretion of IL-1*β*, IL-6, and TNF-*α* proinflammatory cytokines and the expression of NF-*κ*B transcription factor as well as CIC and ACLY—key players of metabolic reprogramming occurring in inflammation—and in turn ROS, NO, and PGE_2_ levels in lipopolysaccharide-triggered macrophages.

## Data Availability

The data used to support the findings of this study are available from the corresponding authors upon request.

## Supplementary Materials

Supplementary Materials Figure S1: effect of *P. lentiscus* hydrosol on primary human monocyte cell viability. Figure S2: effect of *P. lentiscus* hydrosol on U937 cell viability.

## References

[1] Mozaffarian V. (2005). *Trees and Shrubs of Iran*.

[2] Bonnier G., Douin R. (1990). *La Grande flore en couleurs*.

[3] Di Castri F., Goodall D. W., Specht R. L. (1981). *Mediterranean-type shrublands*.

[4] Dellai A., Souissi H., Borgi W., Bouraoui A., Chouchane N. (2013). Antiinflammatory and antiulcerogenic activities of Pistacia lentiscus L. leaves extracts. *Industrial Crops and Products*.

[5] Vlastos D., Mademtzoglou D., Drosopoulou E. (2013). Evaluation of the genotoxic and antigenotoxic effects of Chios mastic water by the in vitro micronucleus test on human lymphocytes and the in vivo wing somatic test on Drosophila. *PLoS One*.

[6] Iauk L., Ragusa S., Rapisarda A., Franco S., Nicolosi V. M. (2013). *In Vitro* Antimicrobial activity ofPistacia lentiscusL. extracts: preliminary report. *Journal of Chemotherapy*.

[7] Magiatis P., Melliou E., Skaltsounis A. L., Chinou I. B., Mitaku S. (1999). Chemical composition and antimicrobial activity of the essential oils of *Pistacia lentiscusvar*.*chia*. *Planta Medica*.

[8] Koutsoudaki C., Krsek M., Rodger A. (2005). Chemical composition and antibacterial activity of the essential oil and the gum of Pistacia lentiscus var. chia. *Journal of Agricultural and Food Chemistry*.

[9] Longo L., Scardino A., Vasapollo G. (2007). Identification and quantification of anthocyanins in the berries of Pistacia lentiscus L., Phillyrea latifolia L. and Rubia peregrina L. *Innovative Food Science & Emerging Technologies*.

[10] Bhouri W., Derbel S., Skandrani I. (2010). Study of genotoxic, antigenotoxic and antioxidant activities of the digallic acid isolated from Pistacia lentiscus fruits. *Toxicology In Vitro*.

[11] Azaizeh H., Halahleh F., Abbas N. (2013). Polyphenols from Pistacia lentiscus and Phillyrea latifolia impair the exsheathment of gastro-intestinal nematode larvae. *Veterinary Parasitology*.

[12] Dedoussis G. V., Kaliora A. C., Psarras S. (2004). Antiatherogenic effect of Pistacia lentiscus via GSH restoration and downregulation of CD36 mRNA expression. *Atherosclerosis*.

[13] Atmani D., Chaher N., Berboucha M. (2009). Antioxidant capacity and phenol content of selected Algerian medicinal plants. *Food Chemistry*.

[14] Remila S., Atmani-Kilani D., Delemasure S. (2015). Antioxidant, cytoprotective, anti-inflammatory and anticancer activities of Pistacia lentiscus (Anacardiaceae) leaf and fruit extracts. *European Journal of Integrative Medicine*.

[15] Congiu R., Falconieri D., Marongiu B., Piras A., Porcedda S. (2002). Extraction and isolation of Pistacia lentiscus L. essential oil by supercritical CO2. *Flavour and Fragrance Journal*.

[16] Douissa F. B., Hayder N., Chekir-Ghedira L. (2005). New study of the essential oil from leaves of Pistacia lentiscus L. (Anacardiaceae) from Tunisia. *Flavour and Fragrance Journal*.

[17] Barra A., Coroneo V., Dessi S., Cabras P., Angioni A. (2007). Characterization of the volatile constituents in the essential oil of Pistacia lentiscus L. from different origins and its antifungal and antioxidant activity. *Journal of Agricultural and Food Chemistry*.

[18] Benyoussef E. H., Charchari S., Nacer-Bey N., Yahiaoui N., Chakou A., Bellatreche M. (2005). The essential oil ofPistacia lentiscusL. from Algeria. *Journal of Essential Oil Research*.

[19] Maxia A., Sanna C., Frau M. A., Piras A., Karchuli M. S., Kasture V. (2011). Anti-inflammatory Activity of *Pistacia lentiscus Essential Oil*: Involvement of IL-6 and TNF-*α*. *Natural Product Communications*.

[20] Mharti F. Z., Lyoussi B., Abdellaoui A. (2011). Antibacterial activity of the essential oils of Pistacia lentiscus used in Moroccan folkloric medicine. *Natural Product Communications*.

[21] *Essential Oils Market Size, Share & Trends Report Essential Oils Market Size, Share & Trends Analysis Report By Application(Food & Beverages, Spa & Relaxation), By Product (Orange, Peppermint), By Sales Channel, And Segment Forecasts, 2020 - 2027*.

[22] Rajeswara Rao B. R. (2012). Hydrosols and water-soluble essential oils of aromatic plants: future economic products. *Indian Perfumer*.

[23] Sumner L. W., Mendes P., Dixon R. A. (2003). Plant metabolomics: large-scale phytochemistry in the functional genomics era. *Phytochemistry*.

[24] Ventura G., Calvano C. D., Losito I. (2019). Effect of pH and mobile phase additives on the chromatographic behaviour of an amide-embedded stationary phase: cyanocobalamin and its diaminemonochloro-platinum(II) conjugate as a case study. *Journal of Separation Science*.

[25] Pascale R., Acquavia M. A., Cataldi T. R. I. (2020). Profiling of quercetin glycosides and acyl glycosides in sun-dried peperoni di Senise peppers (Capsicum annuum L.) by a combination of LC-ESI(-)-MS/MS and polarity prediction in reversed-phase separations. *Analytical and Bioanalytical Chemistry*.

[26] Bianco G., Zianni R., Anzillotta G. (2013). Dibenzo-p-dioxins and dibenzofurans in human breast milk collected in the area of Taranto (Southern Italy): first case study. *Analytical and Bioanalytical Chemistry*.

[27] Bianco G., Agerbirk N., Losito I., Cataldi T. R. (2014). Acylated glucosinolates with diverse acyl groups investigated by high resolution mass spectrometry and infrared multiphoton dissociation. *Phytochemistry*.

[28] Bianco G., Labella C., Pepe A., Cataldi T. R. (2013). Scrambling of autoinducing precursor peptides investigated by infrared multiphoton dissociation with electrospray ionization and Fourier transform ion cyclotron resonance mass spectrometry. *Analytical and Bioanalytical Chemistry*.

[29] Cataldi T. R., Orlando D., Nardiello D. (2007). A three-factor Doehlert matrix design in optimising the determination of octadecyltrimethylammonium bromide by cation-exchange chromatography with suppressed conductivity detection. *Analytica Chimica Acta*.

[30] Pascale R., Bianco G., Cataldi T. R. I. (2018). Investigation of the effects of virgin olive oil cleaning systems on the secoiridoid aglycone content using high performance liquid chromatography–mass spectrometry. *Journal of the American Oil Chemists' Society*.

[31] Cataldi T. R., Bianco G., Abate S., Losito I. (2011). Identification of unsaturated N-acylhomoserine lactones in bacterial isolates of Rhodobacter sphaeroides by liquid chromatography coupled to electrospray ionization-hybrid linear ion trap-Fourier transform ion cyclotron resonance mass spectrometry. *Rapid Communications in Mass Spectrometry*.

[32] Caniani D., Esposito G., Gori R. (2019). Toward a new plant-wide experimental and modeling approach for reduction of greenhouse gas emission from wastewater treatment plants. *Journal of Environmental Engineering*.

[33] Hughey C. A., Rodgers R. P., Marshall A. G. (2002). Resolution of 11,000 compositionally distinct components in a single electrospray ionization Fourier transform ion cyclotron resonance mass spectrum of crude oil. *Analytical Chemistry*.

[34] Qabaha K., Ras S. A., Abbadi J., Al-Rimawi F. (2016). Anti-inflammatory of both *Eucalyptus* spp. and *Pistascia lentiscus* were investigated along with their phenolic compounds analysis using HPLC with UV detection. *African Journal of Traditional, Complementary and Alternative Medicines*.

[35] Infantino V., Pierri C. L., Iacobazzi V. (2020). Metabolic routes in inflammation: the citrate pathway and its potential as therapeutic target. *Current Medicinal Chemistry*.

[36] Infantino V., Convertini P., Cucci L. (2011). The mitochondrial citrate carrier: a new player in inflammation. *The Biochemical Journal*.

[37] Infantino V., Iacobazzi V., Palmieri F., Menga A. (2013). ATP-citrate lyase is essential for macrophage inflammatory response. *Biochemical and Biophysical Research Communications*.

[38] Wong C. C., Cociorva D., Venable J. D., Xu T., Yates J. R. (2009). Comparison of different signal thresholds on data dependent sampling in Orbitrap and LTQ mass spectrometry for the identification of peptides and proteins in complex mixtures. *Journal of the American Society for Mass Spectrometry*.

[39] Zhurov K. O., Kozhinov A. N., Fornelli L., Tsybin Y. O. (2014). Distinguishing analyte from noise components in mass spectra of complex samples: where to cut the noise?. *Analytical Chemistry*.

[40] Zielinski A. T., Kourtchev I., Bortolini C. (2018). A new processing scheme for ultra-high resolution direct infusion mass spectrometry data. *Atmospheric Environment*.

[41] Pascale R., Bianco G., Cataldi T. R. I. (2018). Mass spectrometry-based phytochemical screening for hypoglycemic activity of Fagioli di Sarconi beans (Phaseolus vulgaris L.). *Food Chemistry*.

[42] Hsu C. S., Qian K., Chen Y. C. (1992). An innovative approach to data analysis in hydrocarbon characterization by on-line liquid chromatography-mass spectrometry. *Analytica Chimica Acta*.

[43] Hughey C. A., Hendrickson C. L., Rodgers R. P., Marshall A. G., Qian K. (2001). Kendrick mass defect spectrum: a compact visual analysis for ultrahigh-resolution broadband mass spectra. *Analytical Chemistry*.

[44] Kendrick E. (1963). A mass scale based on CH2 = 14.0000 for high resolution mass spectrometry of organic compounds. *Analytical Chemistry*.

[45] Lerno L. A., German J. B., Lebrilla C. B. (2010). Method for the identification of lipid classes based on referenced Kendrick mass analysis. *Analytical Chemistry*.

[46] Qi Y., Hempelmann R., Volmer D. A. (2016). Two-dimensional mass defect matrix plots for mapping genealogical links in mixtures of lignin depolymerisation products. *Analytical and Bioanalytical Chemistry*.

[47] Sato H., Nakamura S., Teramoto K., Sato T. (2014). Structural characterization of polymers by MALDI spiral-TOF mass spectrometry combined with Kendrick mass defect analysis. *Journal of the American Society for Mass Spectrometry*.

[48] Infantino V., Dituri F., Convertini P. (2019). Epigenetic upregulation and functional role of the mitochondrial aspartate/glutamate carrier isoform 1 in hepatocellular carcinoma. *Biochimica et Biophysica Acta - Molecular Basis of Disease*.

[49] Bradford M. M. (1976). A rapid and sensitive method for the quantitation of microgram quantities of protein utilizing the principle of protein-dye binding. *Analytical Biochemistry*.

[50] Linn T. C., Srere P. A. (1979). Identification of ATP citrate lyase as a phosphoprotein. *The Journal of Biological Chemistry*.

[51] Migita T., Narita T., Nomura K. (2008). ATP citrate lyase: activation and therapeutic implications in non-small cell lung cancer. *Cancer Research*.

[52] Takeda Y., Suzuki F., Inoue H. (1969). [27] ATP citrate lyase (citrate-cleavage enzyme). *Methods in Enzymology*.

[53] Paraschos S., Magiatis P., Gousia P. (2011). Chemical investigation and antimicrobial properties of mastic water and its major constituents. *Food Chemistry*.

[54] Stasiuk M., Kozubek A. (2010). Biological activity of phenolic lipids. *Cellular and Molecular Life Sciences*.

[55] Hwang J. W., Lee S. J., Kim Y. S. (2012). Purification and characterization of a novel peptide with inhibitory effects on colitis induced mice by dextran sulfate sodium from enzymatic hydrolysates of Crassostrea gigas. *Fish & Shellfish Immunology*.

[56] Millán-Linares M. . C., Millán F., Pedroche J., Yust M. d. M. (2015). GPETAFLR: a new anti-inflammatory peptide from Lupinus angustifolius L. protein hydrolysate. *Journal of Functional Foods*.

[57] Qian G. M., Pan G. F., Guo J. Y. (2012). Anti-inflammatory and antinociceptive effects of cordymin, a peptide purified from the medicinal mushroom Cordyceps sinensis. *Natural Product Research*.

[58] Aldini G., Altomare A., Baron G. (2018). N-Acetylcysteine as an antioxidant and disulphide breaking agent: the reasons why. *Free Radical Research*.

[59] Dini I., Tenore G. C., Dini A. (2008). S-Alkenyl cysteine sulfoxide and its antioxidant properties from Allium cepa var. tropeana (red onion) seeds. *Journal of Natural Products*.

[60] Mukwevho E., Ferreira Z., Ayeleso A. (2014). Potential role of sulfur-containing antioxidant systems in highly oxidative environments. *Molecules*.

[61] Bianco G., Buchicchio A., Cataldi T. R. (2015). Structural characterization of major soyasaponins in traditional cultivars of Fagioli di Sarconi beans investigated by high-resolution tandem mass spectrometry. *Analytical and Bioanalytical Chemistry*.

[62] Bianco G., Pascale R., Carbone C. F. (2018). Determination of soyasaponins in Fagioli di Sarconi beans (Phaseolus vulgaris L.) by LC-ESI-FTICR-MS and evaluation of their hypoglycemic activity. *Analytical and Bioanalytical Chemistry*.

[63] Medzhitov R. (2010). Inflammation 2010: new adventures of an old flame. *Cell*.

[64] Medzhitov R. (2008). Origin and physiological roles of inflammation. *Nature*.

[65] Baek Y. S., Haas S., Hackstein H. (2009). Identification of novel transcriptional regulators involved in macrophage differentiation and activation in U937 cells. *BMC Immunology*.

[66] Ghosh S., Hayden M. S. (2008). New regulators of NF-*κ*B in inflammation. *Nature Reviews Immunology*.

[67] Infantino V., Iacobazzi V., Menga A., Avantaggiati M. L., Palmieri F. (2014). A key role of the mitochondrial citrate carrier (SLC25A1) in TNF*α*- and IFN*γ*-triggered inflammation. *Biochimica et Biophysica Acta (BBA) - Gene Regulatory Mechanisms*.

[68] Iacobazzi V., Infantino V., Castegna A. (2017). Mitochondrial carriers in inflammation induced by bacterial endotoxin and cytokines. *Biological Chemistry*.

[69] Indiveri C., Iacobazzi V., Tonazzi A. (2011). The mitochondrial carnitine/acylcarnitine carrier: function, structure and physiopathology. *Molecular Aspects of Medicine*.

[70] Iacobazzi V., Infantino V., Palmieri F. (2008). Epigenetic mechanisms and Sp1 regulate mitochondrial citrate carrier gene expression. *Biochemical and Biophysical Research Communications*.

[71] Infantino V., Iacobazzi V., De Santis F., Mastrapasqua M., Palmieri F. (2007). Transcription of the mitochondrial citrate carrier gene: role of SREBP-1, upregulation by insulin and downregulation by PUFA. *Biochemical and Biophysical Research Communications*.

[72] Iacobazzi V., Convertini P., Infantino V., Scarcia P., Todisco S., Palmieri F. (2009). Statins, fibrates and retinoic acid upregulate mitochondrial acylcarnitine carrier gene expression. *Biochemical and Biophysical Research Communications*.

[73] Iacobazzi V., Infantino V., Bisaccia F., Castegna A., Palmieri F. (2009). Role of FOXA in mitochondrial citrate carrier gene expression and insulin secretion. *Biochemical and Biophysical Research Communications*.

[74] Infantino V., Castegna A., Iacobazzi F. (2011). Impairment of methyl cycle affects mitochondrial methyl availability and glutathione level in Down's syndrome. *Molecular Genetics and Metabolism*.

[75] Convertini P., Todisco S., De Santis F. (2019). Transcriptional regulation factors of the human mitochondrial aspartate/glutamate carrier gene, isoform 2 (SLC25A13): USF1 as basal factor and FOXA2 as activator in liver cells. *International Journal of Molecular Sciences*.

[76] del Arco A., Morcillo J., Martinez-Morales J. R. (2002). Expression of the aspartate/glutamate mitochondrial carriers aralar1 and citrin during development and in adult rat tissues. *European Journal of Biochemistry*.

[77] Iacobazzi V., Infantino V., Costanzo P., Izzo P., Palmieri F. (2005). Functional analysis of the promoter of the mitochondrial phosphate carrier human gene: identification of activator and repressor elements and their transcription factors. *The Biochemical Journal*.

[78] Convertini P., Menga A., Andria G. (2016). The contribution of the citrate pathway to oxidative stress in Down syndrome. *Immunology*.

[79] Santarsiero A., Leccese P., Convertini P. (2018). New insights into Behçet’s syndrome metabolic reprogramming: citrate pathway dysregulation. *Mediators of Inflammation*.

[80] Gul A. (2015). Pathogenesis of Behçet’s disease: autoinflammatory features and beyond. *Seminars in Immunopathology*.

[81] Padula M. C., Leccese P., Lascaro N. (2020). From structure to function for the characterization of ERAP1 active site in Behçet syndrome. A novel polymorphism associated with known gene variations. *Molecular Immunology*.

[82] Todisco S., Convertini P., Iacobazzi V., Infantino V. (2020). TCA cycle rewiring as emerging metabolic signature of hepatocellular carcinoma. *Cancers*.

[83] Iacobazzi V., Infantino V. (2014). Citrate--new functions for an old metabolite. *Biological Chemistry*.

[84] Aktan F. (2004). iNOS-mediated nitric oxide production and its regulation. *Life Sciences*.

[85] Anrather J., Racchumi G., Iadecola C. (2006). NF-*κ*B Regulates Phagocytic NADPH Oxidase by Inducing the Expression of gp91phox. *Journal of Biological Chemistry*.

[86] Park J. Y., Pillinger M. H., Abramson S. B. (2006). Prostaglandin E2 synthesis and secretion: the role of PGE2 synthases. *Clinical Immunology*.

[87] Hassan S. B., Gali-Muhtasib H., Goransson H., Larsson R. (2010). Alpha Terpineol: A Potential Anticancer Agent which Acts through Suppressing NF-*κ*B Signalling. *Anticancer Research*.

[88] Zheng X.-H., Liu C.-P., Hao Z.-G., Wang Y.-F., Li X.-L. (2017). Protective effect and mechanistic evaluation of linalool against acute myocardial ischemia and reperfusion injury in rats. *RSC Advances*.

[89] Lou H., Kaplowitz N. (2007). Glutathione depletion down-regulates tumor necrosis factor alpha-induced NF-kappaB activity via IkappaB kinase-dependent and -independent mechanisms. *The Journal of Biological Chemistry*.

[90] Rose J. (2001). *375 Essential Oils and Hydrosols*.

[91] Catty S. (2001). *Hydrosols: The Next Aromatherapy*.

[92] Price L., Price S. (2004). Understanding Hydrolats : The Specific Hydrosols for Aromatherapy. *A Guide for Health Professionals*.

[93] Paolini J., Leandri C., Desjobert J. M., Barboni T., Costa J. (2008). Comparison of liquid-liquid extraction with headspace methods for the characterization of volatile fractions of commercial hydrolats from typically Mediterranean species. *Journal of Chromatography A*.

