# Supplementary material for: Pistacia lentiscus Hydrosol: Untargeted Metabolomic Analysis and Anti-Inflammatory Activity Mediated by NF-κB and the Citrate Pathway
Source: Oxid Med Cell Longev. 2020 Nov 1;2020:4264815. doi: 10.1155/2020/4264815 (PMC7652607; doi:10.1155/2020/4264815)
Supplement: Supplementary Materials — Figure S1: effect of P. lentiscus hydrosol on primary human monocyte cell viability. Figure S2: effect of P. lentiscus hydrosol on U937 cell viability. [file 4264815.f1.pdf]

Supplementary Figures

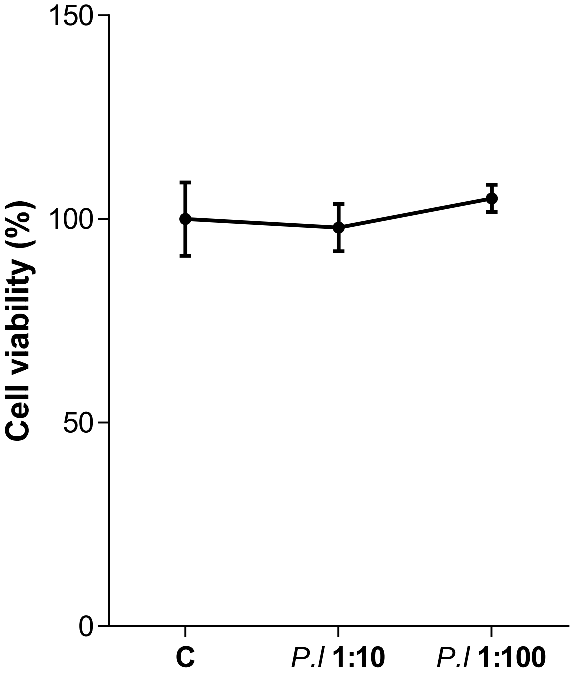

Figure S1. Effect of *P. lentiscus* hydrosol on primary human monocytes cell viability. Primary human $CD14^+$ monocytes were treated with *P.l* 1:10 and *P.l* 1:100. After 72 hours cell viability was assessed by MTT assay. Means ± S.D. of three replicate independent experiments are shown. Differences between treated and untreated cells (C, set at 100%) were not significant (*one-way ANOVA*).

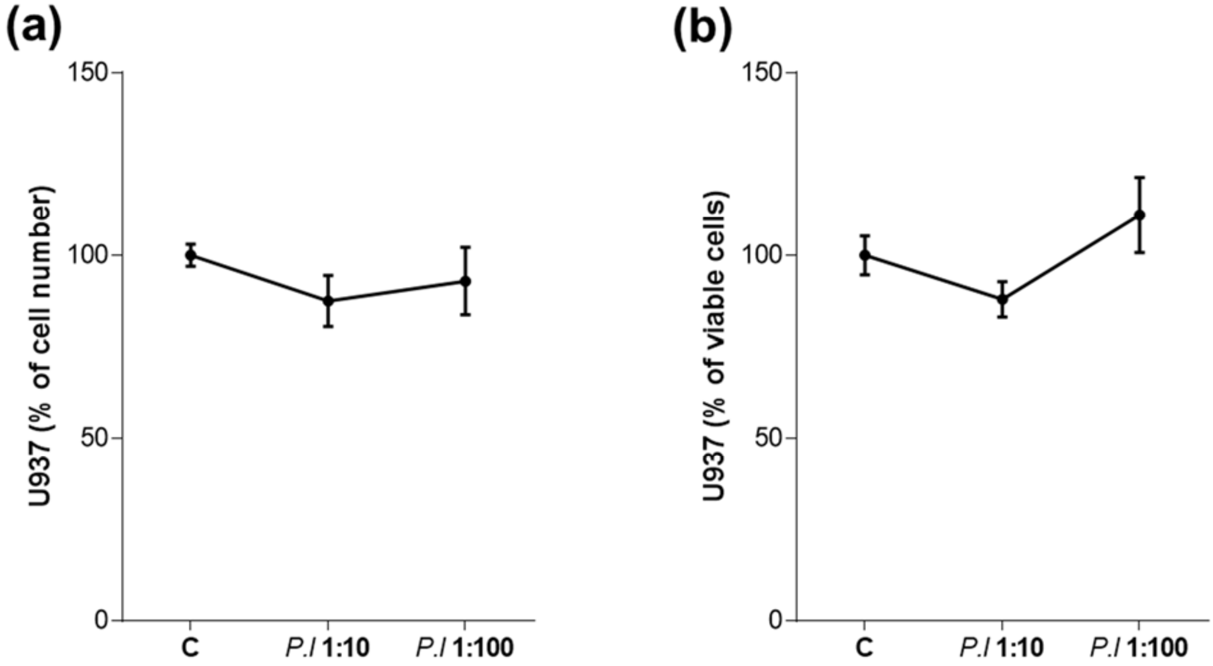

Figure S2. Effect of *P. lentiscus* hydrosol on U937 cell viability. U937 cells were treated with *P.l* 1:10 and *P.l* 1:100. After 72 hours cell viability was assessed by cell counting (a) and MTT assay (b). Means ± S.D. of three replicate independent experiments are shown. Differences between treated and untreated cells (C, set at 100%) were not significant (*one-way ANOVA*).
